# Initial In Vivo Analyses of Small Pore Polymer Scaffolds for Creation of an Artificial Cranial Stem Cell Niche

**DOI:** 10.3390/bioengineering13040420

**Published:** 2026-04-02

**Authors:** Elizabeth Soulas, W. Benton Swanson, Hwa Kyung Nam, Kelsey Gruber, Yuji Mishina, Nan E. Hatch

**Affiliations:** 1 Department of Orthodontics and Pediatric Dentistry, School of Dentistry, University of Michigan, Ann Arbor, MI 48109, USA; esoulas@umich.edu (E.S.); hknam@umich.edu (H.K.N.); klgruber@umich.edu (K.G.); 2 Department of Biologics and Materials Sciences, School of Dentistry, University of Michigan, Ann Arbor, MI 48109, USA; wbentons@umich.edu (W.B.S.); mishina@umich.edu (Y.M.)

**Keywords:** tissue engineering, mesenchymal stem cells, craniosynostoses, skull, biomedical engineering

## Abstract

Craniosynostosis is the premature fusion of skull bones due to loss of stem/progenitor cells located in non-mineralized tissue between growing cranial bones of infants. We generated scaffolds from a biodegradable biomaterial with small interconnected pores (125–250 μm diameter), previously shown to maintain stemness of a mesenchymal cell population, to further develop a method for the creation of an artificial cranial bone stem cell niche. Polymer scaffolds of consistent pore size were fabricated using a molecular-sieved sugar sphere casting technique with poly-l-lactic acid. A rectangular surgical defect within the parietal bone of juvenile mice was created. The three groups included sham animals with surgery but no scaffold, experimental animals with surgery plus an implanted cell-free scaffold, and experimental animals with surgery plus an implanted bone mesenchymal cell-seeded scaffold. Healing at the surgical site was evaluated at 4 and 12 weeks after surgery by micro-CT and histology. Surgical site bone volume fraction and bone mineral density were significantly greater at twelve than four weeks in the sham group but not in either of the scaffold groups. At twelve weeks, the surgical site bone volume fraction and bone mineral density were significantly lower in the cell-seeded scaffold as compared to the sham animal group. At twelve weeks, the anterior and middle cranial vault widths were significantly greater in the cell-seeded scaffold as compared to the sham animal group on the surgery side of the skulls. Less mineralization was evident within the cell-seeded than the cell-free scaffolds by histology. Based on these findings, scaffolds of sufficiently small pore size seeded with autologous bone mesenchymal stem cells could function as an artificial cranial stem cell niche to inhibit surgical-site mineralization and promote cranial growth.

## 1. Introduction

Cranial bones form through the process of intramembranous ossification, whereby osteoblasts differentiate directly from mesenchymal stem cells [1]. Cranial bones grow along their outer edge (known as the osteogenic front), eventually coming into proximity with each other. A cranial suture is formed between the two adjacent growing cranial bone osteogenic fronts. The cranial suture contains mesenchyme tissue that is not mineralized and provides stem and osteoprogenitor cells needed for continued cranial bone growth [2,3]. Craniosynostosis is a condition in which the loss of intervening cranial suture tissue causes fusion of the cranial bones [4]. This can cause high intracranial pressure that can interfere with the normal development of the brain and lead to permanent vision loss and other impairments [5]. Craniosynostosis leads to abnormal skull growth due to the loss of cranial bone growth in areas where the suture tissue is lost, as well as compensating bone overgrowth in areas where the suture tissue remains [6]. The estimated prevalence of craniosynostosis is approximately one in 2000 to one in 2500 live births [7,8], with isolated nonsyndromic craniosynostosis (individuals with no known genetic basis) accounting for approximately 90% of all cases [9,10].

Because the sole treatment for craniosynostosis is surgery, even with appropriately early diagnosis some patients suffer significant morbidity [5,11]. Infants and/or children can also require repeat surgeries that increase the medical and financial burden [12]. These additional procedures are needed to improve skull and facial bone relationships and/or to release post-operative intracranial hypertension. High intracranial pressure occurs unpredictably after surgical correction of craniosynostosis, and when left untreated it is associated with high morbidity [13,14]. While cranial vault remodeling for the surgical correction of craniosynostosis is more invasive and involves greater patient risk, less invasive craniectomy procedures have a higher risk for intracranial hypertension post-surgery [15,16]. Nonsyndromic craniosynostosis most commonly occurs in the sagittal suture and surgical correction of sagittal suture fusion has the highest incidence of post-surgical intracranial hypertension [16,17,18]. Therefore, the goal of this study was to investigate a tissue engineering technique that would be applicable to individuals with craniosynostosis without genetic mutations who may otherwise develop post-surgical morbidities such as limited skull growth and/or cranial suture re-fusion that can result in high intracranial pressure.

Tissue engineering lies at the intersection of science and medicine, aiming to generate functional tissues to better treat a variety of diseases/disorders [19]. Given the risk of repeat cranial bone fusion with intracranial hypertension following an initial surgical intervention in some craniosynostosis patients, and in particular with the more limited craniectomy surgical intervention, it is desirable to implement a tissue engineering approach which will diminish the need for secondary surgeries. Ideally, such an approach would also promote cranial bone growth in a manner similar to that seen with a cranial suture, perpendicular to that suture. Preventing the premature loss of cranial suture stem/progenitor cells necessitates that the suture cell population undergo a controlled, timely differentiation while maintaining a central stem cell population for continued skull growth. Research from our group previously demonstrated that a sugar sphere technique can be used to generate porous nanofibrous PLLA (poly(L-lactic acid)) scaffolds of specific pore sizes [20]. Smaller pore sizes (<250 μm) are key in maintaining stemness of the cell population, while larger pore sizes (>250 μm) enable osteoblast differentiation, matrix maturation and mineralization [21,22]. Smaller pore sizes maintain stemness primarily through the high principal curvature of small pores that prevents cell spreading and promotes cell rounding as well as aggregation. The subsequent change in mechanosensitive signaling that occurs in cells located within small pores creates a stem cell niche region that is maintained in vitro and when implanted subcutaneously in mice [21].

The knowledge gained through this work demonstrating the effect of varying pore sizes on bone mesenchymal stem cell maturation has enabled work to create scaffolds consisting of a specific pore size to control cranial cell stemness and osteogenic differentiation for use in bones. Based upon the concept that a healthy region of non-mineralized tissue between cranial bones containing a population of stem and progenitor cells [2] is required for cranial bone growth, we hypothesize that implanting a scaffold of sufficiently small pore size could function as an artificial cranial stem cell niche to promote cranial bone growth as needed, such as in craniosynostosis patients where the suture stem cell niche is lost. The primary objective of this study was to investigate the utility of small pore size, three-dimensional polymer nanofibrous scaffolds for the prevention of scaffold mineralization and the promotion of cranial bone growth when implanted into the cranial bone surgical sites of mice in a phase of rapid skull growth. A secondary objective of this study was to compare scaffolds that were seeded with bone mesenchymal stem cells with scaffolds that were free of cells prior to implantation to determine if the scaffold alone could be used.

## 2. Materials and Methods

### 2.1. Fabrication of Three-Dimensional Porous Scaffolds

Poly (L-lactic acid) (PLLA, Resomer L207S) with an inherent viscosity of 1.6 dL/g was purchased from Boehringer Ingelheim, Ingelheim am Rhein, Germany; Span80, tetrahydrofuran (THF) and hexane were purchased from Fisher Scientific, Waltham, MA, USA; D-fructose was purchased from Oakwood Chemical; and mineral oil was purchased from Alfa Aesar, Ward Hill, MA, USA. PLLA is established as biocompatible and biodegradable [23]. Nanofibrous scaffolds with specific pore size were fabricated using poly (L-lactic acid) (PLLA) as previously described [20]. PLLA was dissolved in THF solvent at 60 °C. Separately, D-fructose was heated to melt, then emulsified in hot mineral oil with Span80 surfactant by mechanical stirring. The fructose–mineral oil mixture was quenched on ice to solidify the sugar spheres resulting from the emulsion. Sugar spheres were washed with hexane, separated by size using a molecular sieve (Newark Wire Cloth, Cedar Grove, NJ, USA) to control pore size, then loaded into Teflon molds. The small-size sugar spheres were annealed in hexane at 37 °C to cause the spheres to adhere to each other, introducing pore interconnectivity. Hexane was removed under vacuum then PLLA (10% *w*/*v* in THF) was cast and immediately chilled to induce phase separation. After 48 h, the sugar–polymer constructs were transferred to hexane to exchange the THF solvent for an additional 24 h, then soaked in water for 24 h to completely remove the mineral oil/sugar spheres. This process resulted in a nanofibrous, three-dimensional scaffold of consistent and sufficiently small pore size (<250 μm diameter) that could be cut to size before use. Scaffold morphology and consistent pore size following this protocol were previously confirmed by scanning electron microscopy [20].

### 2.2. Scaffold Sterilization

PLLA scaffolds were sterilized by a dual sterilization method. First, the constructs were sterilized using ethylene oxide gas. Second, the scaffolds were washed with 70% ethanol (Millipore Sigma, Burlington, MA, USA) for 30 min, followed by washing with PBS then with cell culture media immediately before cell seeding. The purpose of the 70% ethanol wash is twofold—first, as a secondary sterilization method, and second, to “wet” the surface of the hydrophobic PLLA scaffold prior to cell seeding.

### 2.3. Animal Care and Use

Mice were fed standard lab chow ad libitum with unlimited access to water and housed under a standard 12 h day/night light cycle. Mice were maintained and used in compliance with institutional animal care protocols of the University of Michigan University Committee on Use and Care of Animals (protocol PRO00012117, expiration 19 November 2027), and in accordance with federal guidelines for use and care of animals in research. Timed pregnant C57BL/6J mice were purchased from Jackson Laboratory (Bar Harbor, ME, USA) to generate 3-week-old mice for surgeries +/− scaffold implantations. This age of mice was chosen due to the fact that the rate of skull growth at this age is a phase of rapid growth [24]. Because all the animals remained healthy and viable, no animals were excluded from the study. Sexes were combined for analyses. The experimental unit for in vivo studies was one mouse. Sample sizes for 4- and 12-week post-surgery +/− scaffold +/− cell implantation groups were n = 6. Primary endpoints were the animal health and survival post-surgery, the analysis of mineralization within the surgical site, and the skull morphology measurement. The secondary endpoint was the histology of the surgical site containing scaffolds.

#### 2.3.1. Isolation of Primary Cells from Mice and Scaffold Cell Seeding

C57BL/6J mice were used for the bone mesenchymal stem cell isolation to minimize the potential immune response to implanted cells. Bone mesenchymal stem cells (BMSCs) were isolated from femurs of 14-day-old mice, as previously described [25]. Briefly, epiphyseal growth plates were removed, and the marrow was collected by flushing with media using a 5 mL syringe and 22-gauge needle until the bone appeared white, followed by cell culture in custom formulation αMEM media containing no ascorbate supplemented with antibiotics and fetal bovine serum. Suspension cells were removed every several days via media changes until all suspension cells were gone and adherent cells were just confluent. Cells were seeded at 1.0 × 10^5^ cells/scaffold onto each side of the scaffold (approximately 5 mm length × 1.5 mm width) by gentle pipetting. Cells were allowed to adhere for 30 min. Culture media was then gently added to fully cover the cell-seeded scaffolds. Cells were allowed to attach in the culture for an additional 24 h prior to surgical implantation into mice.

#### 2.3.2. Murine Cranial Bone Surgery and Scaffold Implantation

**Figure 1:**
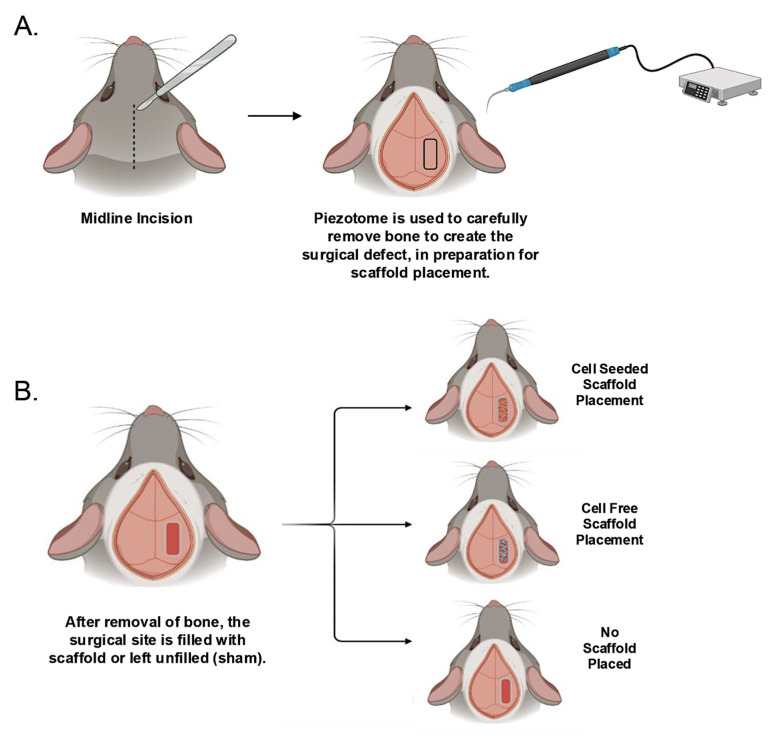
Cranial bone surgical defect creation and scaffold placement procedures. (**A**) A surgical defect of approximately 1.5 mm × 5 mm was created in the parietal bone, after midline cutaneous incision and retraction. (**B**) A small-pore scaffold that had been seeded with bone mesenchymal stem cells, or a scaffold that was not seeded with cells, or no scaffold, was placed into the surgical defect. All mice tolerated the procedure well, remained healthy, and were utilized in subsequent analyses.

Mice were anesthetized via isoflurane inhalation and a midsagittal incision was made on the dorsal aspect of the cranium of each mouse to raise a skin flap. A piezotome was used for the cranial osteotomy (Piezotome Flex, Mectron, Genova, Italy) with saline irrigation. Settings for the surgery were power 3–4, irrigation 1 and mode 2. On one side of the sagittal suture an approximately 1.5 mm width × 5 mm length surgical defect site within the parietal bone was created. This is a critical size defect that will not heal on its own. Surgical sites were either left unfilled (sham group), filled with a cell-seeded scaffold, or filled with a cell-free scaffold (Figure 1). All scaffolds were gently handled with a microspatula to preserve integrity upon insertion into the surgical site. Incisions were closed with several simple interrupted sutures using monofilament absorbable suture material. Animals were given analgesic medication (carprofen) to manage pain. Mice were monitored closely and showed no adverse signs after the implantation surgery. At 4- and 12-week time points following surgery, mice were sacrificed by CO_2_ inhalation overdose followed by bilateral pneumothorax. Mouse skulls were serially dehydrated, fixed in paraformaldehyde, scanned by nano-computed tomography (nano-CT), then embedded in paraffin for histology.

### 2.4. Nano-Computed Tomography (CT)

Whole mouse skulls were scanned using a nanotom M nano-CT system (Waygate Technologies, Baker Hughes, Hürth, Germany). The X-ray tube was powered to 80 kV and 400 µA, with a diamond-coated tungsten target and a 0.381 mm aluminum filter. Imaging was carried out at 12 µm voxel size using an exposure time of 500 ms. Image acquisition and reconstruction of raw data were performed using Datos|x 2 version 2.6.1 (Waygate Technologies). 3D data was reconstructed at 18 voxel resolution for bone analyses. Nano-CT files were re-oriented to all be in the same head position. The 3D nano-CT files were analyzed using Dragonfly image analysis software (Version 2021.1.0.977; Object Research Systems, Montreal, QC, Canada) for bone mineral density (BMD), tissue mineral density (TMD) and bone volume fraction (BVF). These parameters were deemed most meaningful because these measurements are normalized parameters, and because the distinction between trabecular and cortical bone is not yet evident in juvenile murine cranial bones. A region of interest (ROI) was custom drawn around each surgically created site using Dragonfly software, limited to the edges and thickness of the surrounding parietal bone and limited to the superior and inferior widths of the surrounding parietal bone (Supplementary Materials Figure S1). The groups analyzed were sham (surgical site created, no scaffold placed), cell-free scaffold (surgical site created with cell-free scaffold placed) and cell-seeded scaffold (surgical site created with cell-seeded scaffold placed).

### 2.5. Cranial Bone Length/Width Analyses

**Figure 2:**
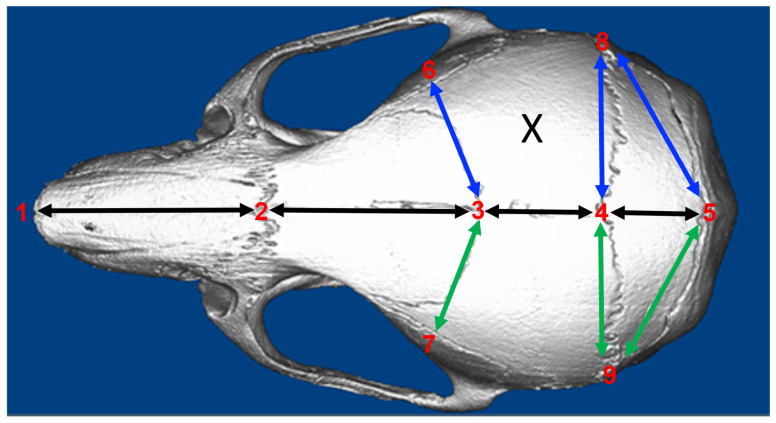
Craniofacial landmarks and measured linear distances. Anterior–posterior measurements made were 1-2: nasal bone length, 2-3: frontal bone length, 3-4: parietal bone length, 4-5: intraparietal bone length. Lateral measurements made were: 3-6 and 3-7, anterior cranial vault widths; 4-8 and 4-9, middle cranial vault widths; and 5-8 and 5-9, posterior cranial vault widths. Black lines: anterior–posterior sagittal skull distances (cranial bone lengths). Blue lines: surgery site side cranial vault widths. Green lines: no surgery site side cranial vault widths. “X” marks the site of cranial bone surgery/defect creation +/− scaffold placement.

To assess changes in cranial bone dimensions between groups and within groups at different time points, linear measurements were performed using a subset of previously established cranial landmarks (Figure 2) [26]. Importantly, most of these landmarks are also present in the human skull and could therefore be used to measure specific aspects of cranial growth with 3D-computed tomographic (CT) images of individuals pre- and post-surgery for craniosynostosis.

3D nano-CT files were downsized to 24-micron resolution and Dolphin Imaging Software version 11.8 (Dolphin Imaging & Management Solutions, Chatsworth, CA, USA) was used to measure linear distances between the following points: 1 (nasale, intersection of the nasal bones, rostral point), 2 (nasion, intersection of the nasal bones, caudal point), 3 (bregma, intersection of frontal bones and parietal bones at the midpoint), 4 (pari, intersection of the parietal and anterior aspect of interparietal bones at the midline), 5 (paro, intersection of the interparietal and occipital bones at the midline), 6 and 7 (bilateral landmarks at frontal-squamosal intersection at temporal crest), 8 and 9 (bilateral landmarks at the joining of squamosal body to zygomatic process of squamous portion of temporal bone), and 10 and 11 (bilateral landmarks at the intersection of parietal, temporal and occipital bones). Groups used for linear skull morphologic analyses were 1) sham (surgical site created, no scaffold placed), 2) cell-free scaffold (surgical site created with cell-free scaffold placed) and 3) cell-seeded scaffold (surgical site created with cell-seeded scaffold placed).

### 2.6. Histologic Preparation and Staining

Implanted scaffolds were carefully removed by dissection then handled using a microspatula for transfer into fixation to preserve integrity of the sample. Samples were fixed using paraformaldehyde then dehydrated in a series of ethanol washes before embedding in paraffin. Serial sections of the parietal cranial bone region containing the surgical site +/− scaffold +/− seeded cells were cut at 5 um thickness in the coronal plane. Standard protocols were used for Masson’s trichrome staining.

### 2.7. Statistical Analysis

All data are reported as mean +/− standard deviation and represent a sample size of n = 6. Statistical analysis was carried out in GraphPad Prism v10. An ANOVA was used to assess differences between all groups, followed by an unpaired t-test with Welch’s correction for direct comparison between individual groups when the data was established as normal. A Mann–Whitney test was used when the data was established as not normal. *p* < 0.05 was considered significant.

## 3. Results

### 3.1. Animal Health

All animals tolerated the surgical procedure well and were deemed healthy (based upon weight gain, coat condition and behavior) up to and until euthanasia.

### 3.2. Nano-CT of the Surgical Site with/Without Scaffold with/Without Cells

Bone mineral density (BMD, a measure of all mineral within a region of interest) was greater in 12-week animals than in 4-week animals only in the sham group. Tissue mineral density (TMD, a measure of the density of mineral that thresholds as bone tissue) was greater in animals at 12 weeks than at 4 weeks in all groups. Bone volume fraction (BVF, a measure of the volume of bone within a region of interest) was greater in animals at 12 weeks than at 4 weeks only in the sham group. Please note that all comparisons beween 4 and 12 weeks compare different groups of animals (animals were euthanized and analyzed at 4 weeks and other animals were euthanized and analyzed at 12 weeks) such that these comparisons between time points are cross-sectional and cannot be interpreted as longitudinal assessments. A comparison of the 4-week and 12-week animals does allow for comparisons based upon the length of time that a surgical defect +/− scaffold was present.

**Figure 3:**
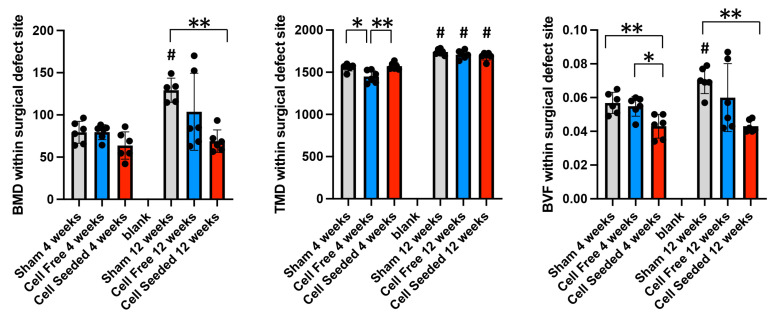
Mineralization in surgical defect site at four and twelve weeks post-surgery. Mouse skulls were analyzed by nano-computed tomography (nano-CT) 4 or 12 weeks after cranial osteotomy +/− scaffold placement +/− cell seeding. Mean +/− standard deviations are shown. # *p* > 0.005 between 4 and 12 weeks; * *p* < 0.05 between indicated groups; ** *p* < 0.005 between indicated groups.

When comparing the groups, the total amount of mineral (BMD) within the defect +/− scaffold +/− cells site was similar in all the groups at 4 weeks post-surgery, while the cell-seeded scaffold group had significantly less mineral (BMD) than the sham group at 12 weeks post-surgery. The BMD for the cell-free scaffold group was variable. TMD was measured as signficantly less in the cell-free scaffold group as compared to either the sham group or cell-seeded scaffold groups at 4 weeks post surgery. At 12 weeks, no differences in TMD between the groups were evident. BVF was measured as significantly less in the cell-seeded scaffold group when compared to either the sham or the cell-free scaffold groups at 4 weeks post-surgery. The BVF was significantly less in the cell-seeded group when compared to the sham group at 12 weeks post-surgery. The BVF for the cell-free scaffold group was variable at 12 weeks post-surgery. Overall, the cell-seeded scaffold group showed less mineral accumulation in the surgical site after surgical site creation and/or scaffold placement when measured by BMD and BVF, as compared to the other groups (Figure 3). The scaffold that was not seeded with cells prior to implantation showed variability in the degree of mineralization, as assessed by BMD and BVF.

### 3.3. Cranial Bone Lengths and Widths

**Figure 4:**
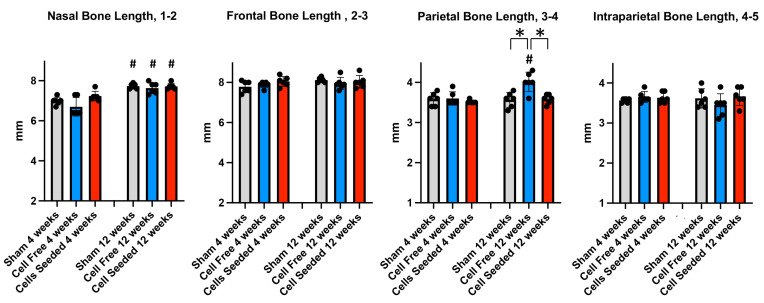
Anterior–posterior skull length measurements. Means +/− standard deviations for nasal, frontal, parietal and intraparietal bone lengths of sham, cell-free scaffold, and cell-seeded scaffold animal groups are shown at 4 and 12 weeks after surgery. # *p* < 0.005 between 4 and 12 weeks post-surgery. * *p* < 0.05 between indicated groups.

The cranial bone lengths and width measurements are shown in Figure 2. For the anterior–posterior skull measurements (Figure 4), the results show that nasal bone length was significantly greater in all the groups at 12 weeks as compared to 4 weeks after surgery +/− scaffold implantation, with no differences between the groups. No significant differences between the groups or time points were found for the anterior–posterior length of the frontal or intraparietal bones. The anterior–posterior length of the parietal bone was significantly greater at 12 compared to at 4 weeks only in the cell-free scaffold group and was also greater in the cell-free as compared to the sham and cell-seeded scaffold groups at 12 weeks after scaffold implantation.

**Figure 5:**
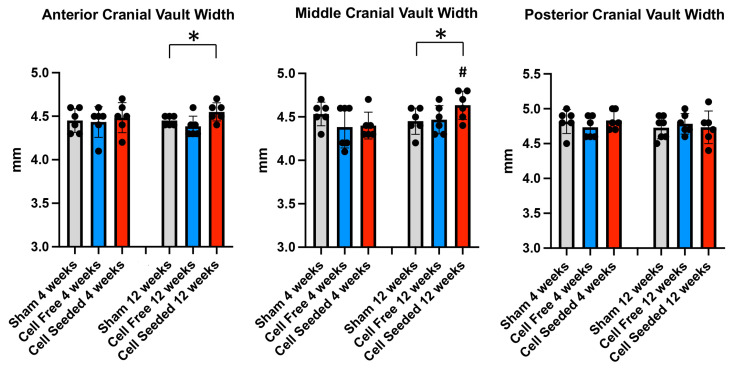
Surgical site side skull width measurements. Means +/− standard deviations for anterior cranial vault, middle cranial vault and posterior cranial vault widths on the side of the skull in which the surgical site was created for sham, cell-free, and cell-seeded animals at 4- and 12-week time points are shown. * *p* < 0.05 between indicated groups, # *p* < 0.05 between time points.

Lateral skull measurements are shown in Figure 5. The anterior and middle cranial vault widths in animals in which a cell-seeded scaffold was placed was significantly greater than in animals in which a cell-free scaffold or no scaffold (sham) was placed at 12 weeks post-surgery. The middle cranial vault width was also greater in animals at 12 weeks than at 4 weeks after surgery only in the cell-seeded scaffold group. No other differences between groups were found.

**Figure 6:**
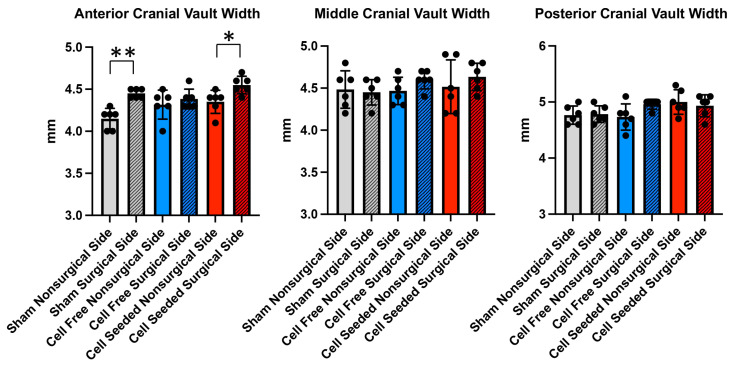
Surgical side vs. nonsurgical side cranial vault width measurements. Mean +/− standard deviations for skull width measurements comparing the side on which the parietal surgical defect was created (surgical side) with the side that had no surgery (nonsurgical side), including measurements of the anterior cranial vault, middle cranial vault and posterior cranial vault, are shown. * *p* < 0.05 and ** *p* < 0.005 between indicated groups.

Lateral skull measurements between the surgical site side and the contralateral/nonsurgical site side are shown in Figure 6. The results show that the anterior cranial vault width was greater on the surgical as compared to the nonsurgical side in the cell-seeded and the sham animal groups. A lot of variation existed in the cell-free scaffold group. No other significant differences between time points or between groups were found.

### 3.4. Histology

**Figure 7:**
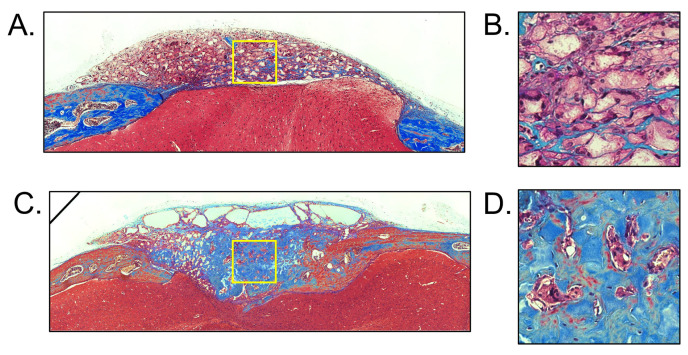
Histology of scaffolds with/without seeded cells at twelve months after implantation. Mason’s Trichome stained coronal sections of the scaffold +/− seeded cells that are surrounded by cranial bone above brain are shown. Cytoplasm stains pink/red; collagen and mineralized collagen stains blue, nuclei stains dark brown/black. (**A**) Bone mesenchymal stem cell (BMSC)-seeded scaffold at 20× magnification, (**B**) magnified area shown in yellow outlined square of (**A**), (**C**) Non-cell-seeded scaffold at 20× magnification, (**D**) magnified area shown in yellow outlined square of (**B**).

Mason’s Trichome staining of the surgical defect site containing the cell-seeded scaffold vs. the cell-free scaffold at 12 weeks after implantation was performed. The results showed the scaffold and tissue present between the intervening parietal bones in animals in which scaffolds were placed (Figure 7). There was no evidence of inflammatory cell infiltrate in any of the samples. Both the cell-free and cell-seeded scaffolds were cellularized, as evidenced by the nuclear staining within the scaffold region. The staining revealed more mineralization (blue stain) of the surgical site when it was implanted with scaffold without cells, as compared to the surgical site when it was implanted with scaffold that was seeded with BMSCs prior to implantation.

## 4. Discussion

The long-term goal of this project is to translate the use of this tissue engineering technique to create an artificial cranial bone stem cell niche in individuals with craniosynostosis or other craniofacial abnormalities where skull growth is needed. For example, a rectangular suture-shaped scaffold that creates a stem cell niche could be provided upon surgical correction of craniosynostosis to prevent re-occurrence, particularly in patients at higher risk for it. Craniosynostosis due to sagittal suture fusion and less invasive lower risk procedures such as craniectomy increase the risk of re-occurrence [15,16,27]. Here, we sought to determine if polymer scaffolds of sufficiently small pore size could be used to create an artificial cranial stem cell niche within cranial bone. We utilized 3-week-old mice because this is an age of rapid skull growth in mice, like that which would be seen in infants undergoing surgical treatment for craniosynostosis or other craniofacial anomalies. We utilized PLLA scaffolds with pores of 125–250 μm because this pore size was previously shown to maintain cells in a stem-like state [21].

That the small-pore polymer scaffold functioned as a cranial bone stem cell niche is evidenced by the fact that the mice remained healthy after implantation, no inflammatory infiltrate was seen, and the surgical site did not mineralize when the scaffold was seeded with stem cells prior to implantation. The lack of mineralization indicates that the cells did not differentiate into osteoblasts. The scaffold that was not seeded with cells prior to implantation did not consistently inhibit mineralization by 12 weeks after implantation. This finding indicates that the scaffold alone may not be fully adequate to control the fate of endogenous cells entering the scaffold, an important finding for future studies. It must be noted that this study included only six animals per group. Given the variability in these results, particularly of the scaffold without cells, future studies must include a greater number of animals per group to definitively determine if this is the case. A greater sample size would also allow for a comparison of the effects between male and female mice.

Importantly, the anterior and middle cranial vault widths on the surgical defect +/− scaffold side were greater in the animals that received cell-seeded scaffolds than the sham-treated animals, indicating greater lateral skull growth in these mice than in the other groups. The middle cranial vault width on the surgical defect +/− scaffold side was also greater at 12 than at 4 weeks only in the cell-seeded scaffold animals. This growth is perpendicular to the shape of the scaffold, similar to that which occurs across an endogenous cranial suture [6]. These results support the idea that the cell-seeded scaffold functioned as an artificial cranial bone cell niche, like that of a cranial suture.

Direct comparison of the surgery with the non-surgery side showed that the anterior cranial vault width on the surgery side was significantly greater than the non-surgery side in both the cell-seeded scaffold and no-scaffold-implanted animals. These results could indicate that the surgery itself promoted skull growth perpendicular to the rectangular surgical defect. The middle cranial vault width measurements between the two skull sides were not significantly different in the cell-seeded scaffold animals, indicating that the surgery alone did not impact this aspect of the skull. Greater variation was found for this middle cranial vault width, again indicating the need for a greater sample size in future studies.

The findings that the anterior and middle cranial vault widths were greater on the surgical side in scaffolds that were seeded with cells (Figure 5), combined with the finding that only the anterior cranial vault width was greater on the surgery than non-surgery side (Figure 6), could be explained by the ability of surgery to impact skull growth across one but not two sutures, while the cell-seeded scaffold impacted skull growth across one and across two cranial sutures. If the surgery enhanced growth across one suture (the sagittal suture), this would account for the greater middle but not anterior cranial vault width in these animals. If the surgery was unable to enhance skull growth across two sutures (the sagittal and coronal sutures), this would account for the lack of greater anterior cranial vault width in these animals. In contrast, if the cell-seeded scaffold enhanced growth across both one and across two sutures, this would account for the finding that both the anterior and middle cranial vault widths were greater on the surgery side in animals that received these scaffolds.

Poly (L-lactic acid) (PLLA) is widely used in tissue engineering [23]. Relevant to this study, Gupte et al. developed a method for generating PLLA scaffolds of specific interconnected pore sizes (very small 60–125 μm, small 125–250 μm, medium 250–425 μm, large 425–600 μm) using the sugar sphere technique described here [20]. These earlier studies showed that large-pore but not small-pore scaffolds enabled vascularization, cell differentiation and cartilage formation when the scaffolds were seeded with cells and cultured in chondrogenic media prior to subcutaneous implantation into mice. Subsequent work confirmed that vascularization was significantly greater in the large-pore compared to small-pore scaffolds, and that mineralization only occurred in the large-pore scaffolds, when stem cell-seeded scaffolds were implanted subcutaneously in mice [22]. Gene expression data from the subcutaneously implanted scaffolds showed that small-pore scaffolds maintained stem cell gene expression, while large-pore scaffolds exhibited osteoblastic gene expression.

The field of craniofacial surgery that attempts to relieve high intracranial pressure and normalize craniofacial growth in infants and toddlers with craniosynostosis has a need to eliminate fusion between prematurely fused cranial bones, decrease intracranial pressure, prevent recurrence of fusion and intracranial pressure, and promote bone growth in a direction perpendicular to that of the previously lost cranial suture. Cranial suture tissue contains a population of undifferentiated stem cells surrounded by progenitor cells that differentiate into osteoblasts [2]. Gli1 is a marker for suture mesenchymal stem cells [3]. Relevant to this study, Gli1+ mesenchymal stem cells regenerated the cranial suture after surgical resection and rescued craniosynostosis-associated cognitive deficits in *Twist*+/− mice when delivered in a methacrylated gelatin/collagen mix [28]. Other work demonstrated that provision of Axin2+ cells, another marker of suture stem cells, can rescue coronal cranial suture fusion in *Twist*+/− mice [29]. These studies are important advances for ultimate translation in the treatment of craniosynostosis in humans, particularly in individuals with mutations in *Twist*. These studies and others have shown that craniosynostosis occurs when cranial suture stem cells are lost prematurely. A limitation of the current study for translational application to individuals with syndromic craniosynostosis is that the studies were performed in wild-type mice. Future studies are required to determine if the small-pore scaffolds can create and maintain a cranial suture niche in an animal model of craniosynostosis, because that environment may impact scaffold efficacy in individuals with syndromic craniosynostosis.

We chose to test PLLA small-pore scaffolds with and without wild-type autologous stem cells in wild-type mice to investigate the ability to create a cranial bone stem cell niche, e.g., an artificial cranial suture, that is more generalizable to a variety of craniosynostosis and other craniofacial anomaly conditions. To our knowledge, this is the first report of a scaffold-based creation of an artificial cranial bone stem cell niche in wild-type mice. Sagittal suture fusion is a relatively common form of craniosynostosis, particularly in nonsyndromic patients with no known mutations [18,30], and has a greater risk of high intracranial pressure after surgical repair than that of other cranial sutures [16,17]. The placement of a polymer-based artificial stem cell niche during surgery could promote skull growth post-surgery and therefore decrease the incidence of recurring high intracranial pressure following sagittal suturectomy. In future studies it will be important to surgically resect the sagittal suture followed by scaffold placement in wild-type mice to fully establish that the small-pore scaffold seeded with BMSCs can function as a replacement sagittal suture. This was attempted at the initiation of this study, but we found challenges with residual bone left in the surgical defect side. Because it is important to test scaffolds in an animal model with a rapidly growing skull, it may be necessary to utilize a larger animal model such as rabbits in future studies to test the scaffold within the sagittal suture.

## 5. Conclusions

This manuscript describes the development of a surgical and scaffold-based procedure for the creation of an artificial cranial stem/progenitor cell niche. Here, we demonstrate that scaffolds composed of interconnected pores of sufficiently small size (125–250 μm diameter) may be sufficient to prevent mineralization for up to 3 months when implanted into cranial bone surgical sites of 3-week-old mice. Scaffolds that were seeded with bone mesenchymal cells prior to implantation were more consistent in this effect. That the small-pore, cell-seeded scaffolds functioned as a cranial stem cell niche was also evidenced by enhanced skull growth perpendicular to the rectangular-shaped surgical defect/scaffold shape, as would be expected to result from an actual cranial suture. With further optimization, small-pore polymer scaffolds hold promise for preventing cranial bone re-fusion in individuals undergoing surgical correction of craniosynostosis using the less invasive and lower morbidity strip suturectomy procedure.

## Data Availability

The raw data supporting the conclusions of this article will be made available by the authors on request.

## Supplementary Materials

The following supporting information can be downloaded at https://www.mdpi.com/article/10.3390/bioengineering13040420/s1: Figure S1. Nano-CT Region of Interest for Bone Mineral Analyses.

## Abbreviations

The following abbreviations are used in this manuscript:

BMSC	Bone mesenchymal stem cell
BMD	Bone mineral density
TMD	Tissue mineral density
BVF	Bone volume fraction
PLLA	poly (L-lactic acid)
THF	Tetrahydrofuran
