# Supplementary material for: Initial In Vivo Analyses of Small Pore Polymer Scaffolds for Creation of an Artificial Cranial Stem Cell Niche
Source: Bioengineering (Basel). 2026 Apr 2;13(4):420. doi: 10.3390/bioengineering13040420 (PMC13113054; doi:10.3390/bioengineering13040420)

Supplemental Figures

Supplemental Figure 1. Nano-CT Region of Interest for Bone Mineral Analyses. A 2D photo of the region of interest containing the cranial bone osteotomy is shown within the Dragonfly analysis software (Version 2021.1.0.977; Object Research Systems) for bone mineral density (BMD), tissue mineral density (TMD) and bone volume fraction (BVF) analyses. Note the custom region of interest of the osteotomy site in the lower left and lower right windows that is colored a combination of light blue, pink and yellow. This software allows for visualization of all three planes of space simultaneously to ensure that an accurate region of interest is being used for the analyses.

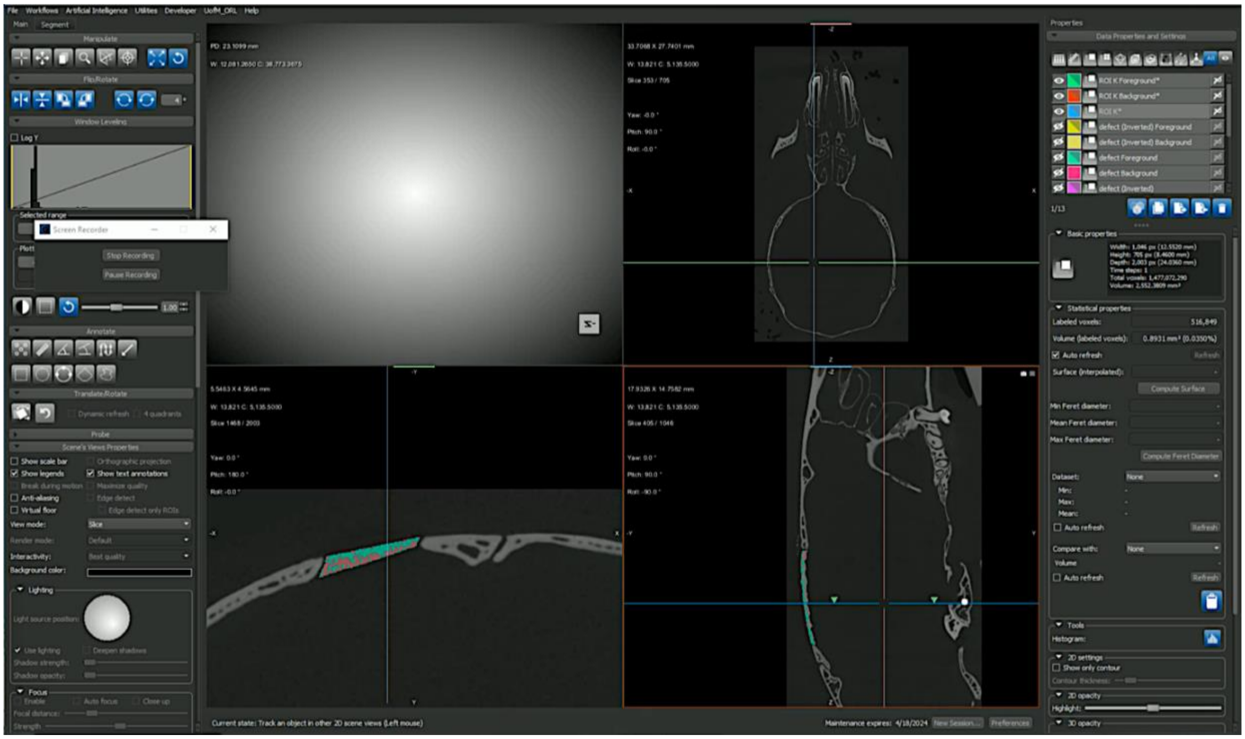

Supplement: Supplementary file 1 [file bioengineering-13-00420-s001.zip › bioengineering-4159173-supplementary.pdf]
